# Potential tree species for use in urban areas in temperate and oceanic climates

**DOI:** 10.1016/j.heliyon.2016.e00154

**Published:** 2016-09-22

**Authors:** Miklas Scholz, Vincent C. Uzomah, Furat A.M. Al-Faraj

**Affiliations:** a Division of Water Resources Engineering, Faculty of Engineering, Lund University, P.O. Box 118, 22100 Lund, Sweden; b Civil Engineering Research Group, School of Computing, Science and Engineering, The University of Salford, Newton Building, Salford M5 4WT, England, United Kingdom

**Keywords:** Engineering, Environmental science

## Abstract

This study aims to assess the potential of trees for integration in urban development by evaluating the damage caused by trees in relation to various tree characteristics. Tree damage to permeable pavement systems and other urban structures such as impermeable pavements, kerbs, roads, retaining walls, footpaths, walls and buildings were assessed to identify the most suitable trees for the urban environment. One hundred square sites of 100 m × 100 m were randomly selected in Greater Manchester for this representative example case study to demonstrate the assessment methodology. Among tree species in this study, *Acer platanoides* L. (Norway maple) occurred most frequently (17%); others were *Tilia spp.* L. (Lime; 16%), *Fraxinus excelsior* L. (common ash; 12%), *Acer pseudoplatanus* L. (sycamore; 10%) and *Prunus avium* L. (wild cherry; 8%). The study concludes that 44% of the damage was to impermeable pavements and 22% to permeable pavements. Other damage to structures included kerbs (19%), retaining walls (5%), footpaths (4%), roads (3%) and walls (3%). Concerning the severity of damage, 66% were moderate, 21% light and 19% severe. *Aesculus hippocastanum* L. (horse chestnut) caused the greatest damage (59%) expressed in percentage as a ratio of the tree number related to damage over the corresponding tree number that was found close to structures.

## 1 Introduction

### 1.1 Rationale

Trees play major roles in creating healthy urban ecosystems and sustainable environments. However, some trees may cause damage to urban structures such as permeable pavements, impermeable pavements, kerbs, roads, footpaths, buildings and retaining walls. For the purpose of this study, permeable pavements are defined as a sustainable system comprising a base and subbase allowing the movement of storm water through the surface, reducing runoff.

Randrup et al. (2003) indicated that in some cities substantial amount of money has been allocated to address conflicts between the rooting system of trees and urban infrastructure. An assessment of this sort becomes important as part of a decision support tool for the fitting and retrofitting of sustainable drainage systems (SuDS), and in the planning of tree planting projects at urban development sites, regeneration projects, and sustainable drainage projects (Scholz and Uzomah, 2013). A SuDS is designed to lessen the potential impact of construction developments with concerning surface water drainage discharges (Scholz, 2010, Scholz, 2015).

### 1.2 Brief literature review

Table 1 summarised tree development characteristics (Pliûra and Heuertz, 2003; Defra, 2007; Arbor Day Foundation, 2015; British Hardwood Tree Nursery, 2015; Garden Centre, 2015). The presence of urban trees often increases property prices (Sander et al., 2010; Scholz, 2010, Scholz, 2015). Trees may be regarded as desirable by residents, because they increase the aesthetic value of a place, and provide ecosystem services including amenities (Scholz and Uzomah, 2013).

The root system of vegetation such as most trees provides the essential functions of anchorage (known as structural stability), absorption of runoff water and nutrients as well as storage of vital food reserves (Scholz, 2010, Scholz, 2015). Tree roots may cause damage to underground utility services by direct pressure on conduits as roots grow and expand in diameter, or by entry to hydraulic services such as sewers and storm water pipes occasionally causing destruction and more frequently blockage (Mather and Morton, 2008). Tree species that have large and vigorous root systems in terms of their growth rate may result in significant destruction of public infrastructure elements including roads, kerbs, footpaths, paved areas and underground utility services. Trees with these characteristics should preferably be avoided or at least controlled. Mechanical forces exerted by radial growth of tree roots can lift relatively light structures such as paths, curbs, paving slabs and boundary walls as well as occasionally single story buildings including porches and garages (Biddle, 1998; Mather and Morton, 2008). However, poor construction of pavements can also cause structural failure (Sydnor et al., 2000).

Some research studies have found strong correlations between the size of specific trees and serious conflicts with infrastructure demands (Mather and Morton, 2008). Large trees usually cause more conflicts than small trees. Damage to pavements correlate with nearby tree diameters. Most trees are linked to damage when they are between 11 and 20 cm in diameter (measured at breast height). However, most Quercus spp. (Oak) and A. hippocastanum do not cause harm, unless they are greater than 20 cm in tree diameter at breast height (DBH) according to Randrup et al. (2003).

Randrup et al. (2003) pointed out that a concrete or asphalt pathway can act as a barrier preventing soil moisture loss by evaporation. This artificial evaporation barrier creates a more humid environment on the underside of the pavement surface, because of temperature differences between the soil and the above pavement. Tree roots are therefore naturally attracted to the condensation water at the soil and impermeable pavement interface (Randrup et al., 2003). This may eventually lead to pavement surface destruction through the radial forces generated during tree root growth.

When tree roots encounter dense soil layers, they usually change direction, stop growing, or adapt by remaining unusually close to the surface. This superficial rooting makes urban trees more vulnerable to drought and can cause destructive pavement heaving (Randrup et al., 2003). The highly compacted soils commonly required for constructing pavements do not allow tree root penetration (Scholz, 2013).

Viswanathan et al. (2011) undertook a research study concerned with the performance of Liquidambar styraciflua L. (American sweetgum) roots under permeable and impermeable pavements. Their results suggested that the standing live root lengths for the American sweetgum were longer in impermeable concrete than in permeable concrete for the first 0 to 20 cm of soil depth. Beyond this depth, the standing live roots were more abundant in permeable than in impermeable pavements. However, they came to the conclusion that pervious concrete does not give a quantifiable root production benefit in comparison to impervious concrete.

Giuliani et al. (2015) used modelling tools to analyse tree growth in street pavements. The findings indicate the progressive reduction of deformations with the increase of the depth of root penetration. However, these studies require a lot of data for individual trees and sites.

### 1.3 Aim, objectives and significance

This study aims to provide a simple method to assess the damage caused by urban tree roots in relation to the corresponding tree characteristics such as species, distance from structures, DBH, tree height, crown spread (diameter), and tree hang-over characteristics. The outcomes should be used to focus on planting the most suitable tree species near specific urban structures in the future.

The objectives are (1) to outline a method for random selection of representative sites in Greater Manchester to study the tree damage characteristics; (2) to identify the predominant trees causing damage to urban structures in Greater Manchester (example case study); and (3) to define a rapid methodology to assess the damage to structures such as permeable pavements, impermeable pavements, roads, kerbs, footpaths, and retention walls for which individual tree species are responsible for. This study provides valuable information for the retrofitting of structures such as permeable pavements in combination with existing trees and to developers in choosing the most suitable trees for the right urban environment minimising damage.

## 2 Methodology

### 2.1 Site selections

**Fig. 1:**
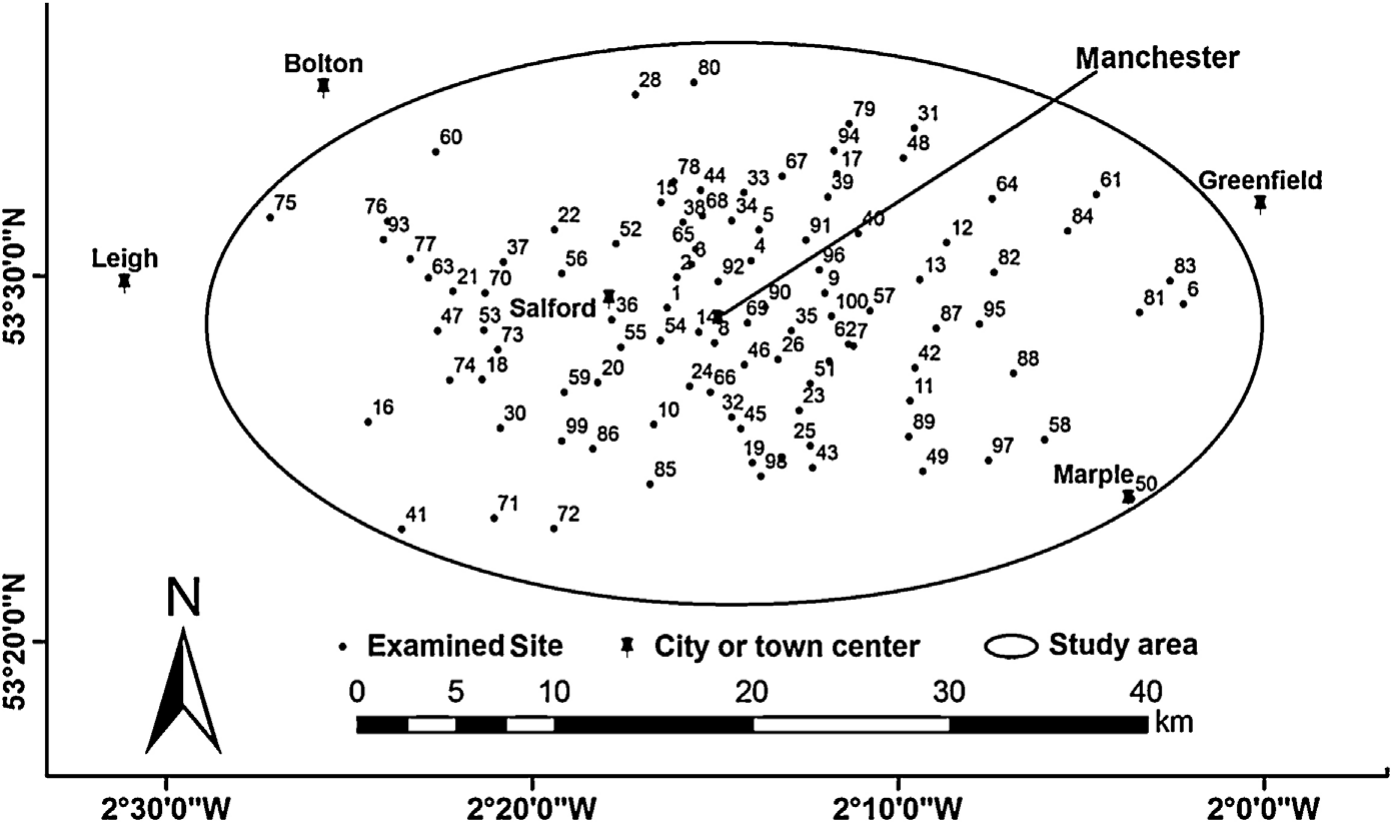
Overview of the assessed case study sites located in the Greater Manchester area.

In order to address objective (1), a total of 100 sites were randomly selected in the Greater Manchester area (North-west England) using the Google Earth map and tools, but restricted within the area of an ellipse covering the main urban areas around Manchester city centre for ease of assessment and to reduce transportation costs (Fig. 1). A square of 100 m × 100 m was drawn around the centre of each selected site to identify an outer boundary for the tree assessment studies. The coordinates, grid references, longitudes, latitudes and post codes of all sites were subsequently determined. The Greater Manchester area is located between 53°28′0″N and 2°14′0″W. The estimated population of Greater Manchester is around 2,680,000.

### 2.2 Tree damage data collection

Data related to tree damage assessments were collected to address objective (2). The data set included variables such as site number, tree number, tree species, common name and genus, tree DBH (1.5 m from ground level), estimated tree height, estimated tree crown diameter, structures near the tree, distance (no maximum threshold) of all aboveground nearby structures from the tree (defined at species level where possible), type of damage (if any) to structures and their severity as well as subjective aesthetic considerations. Site visits were carried out during 2013 and 2014. Predominantly summer periods were chosen because during these periods, trees have their full leaf canopies, which makes tree identifications and corresponding crown spread determinations easier.

All trees within the marked 100 m × 100 m boundaries with a DBH of greater than 10 cm were assessed, except where a site was inaccessible for a valid reason; e.g., some relatively small areas within restricted (private) access areas such as gated private gardens were not assessed, and were subsequently marked as inaccessible sites. Other sites without any tree data entries were without any trees, had only trees where the DBH was less than 10 or were predominantly of a different land use category (without trees) such as water.

The DBH was calculated by measuring the circumference at breast height using a tape measure, and dividing the value by π (approximately 3.14159). Trees less than 10 cm in diameter were not recorded as they were considered too young to cause any measureable damage. Tree heights were estimated using methods based on goniometry (Vernier, 2013), and also by comparing the tree height with nearby structures such as houses as well as electric and telephone poles. Goniometry involves walking away from the base of the trunk until the observer sees the top of the tree from an angle of 45° (which the observer can check using his or her arm). The height of the tree roughly equates to the distance from the tree to where the observer is standing plus his or her eye height from the ground.

### 2.3 Tree damage assessment method

In order to address objective (3), the information and data collected and processed to show the methodology to assess damage by trees is summarised in Table 2, Table 3, Table 4, Table 5, Table 6. Table 2 shows a summary of the most frequently occurring trees and their corresponding damage to urban structures. The structures that were considered in this assessment were permeable pavements, impermeable pavements, kerbs, roads, retaining walls, buildings and footpaths. The footpath structure refers to a walkway though areas such as parks. The damage that was taken into account is lifting-up of structures, disjointing of structures by roots, sinking-in (depression) of structures and cracking-up of structures. The assessment was undertaken with care to distinguish between damage due to trees and/or poor construction (Sydnor et al., 2000). However, all assessments were based on civil engineering expert opinion considering that no disruptive and/or destructive tests could be undertaken on private and public land. Pictures of actual root damage were taken and analysed.

The severity of damage was determined by assigning numbers between 1 and 3, where 1 represents an emerging damage at an early stage (‘light damage’), 2 indicates a damage that is gradually advancing or already well-established (‘moderate damage’), and 3 equates to ‘severe damage’, which is an advanced damage (e.g., pavements completely separated or kerbs completely disjointed) or a well-advanced damage that has become a safety hazard to users requiring immediate attention (or a damage that has already been repaired). It follows that essentially a rather coarse three-category damage scale (Table 3) has been used to reflect the fact that damage to structures by trees is rare despite the large data set collected. Furthermore, the absolute majority of trees did not cause any damage, and could be seen as the control group, which was assigned 0 (no damage recorded).

Table 4 and Table 5 indicate the proportion of tree species that caused structural damage and the relative tree rankings concerning the structural damage, respectively (see Section 3.4 for detailed descriptions and interpretations). Weighting factors reflect the relative importance of structures to the local infrastructure (key criterion of assessment), and have been determined by civil engineering expert judgements expressed by the authors (Table 5). For example, damage to a building receives a higher weighting than damage to pavements. However, pavements are more important than their corresponding kerbs. Finally, Table 6, which has also been informed by the literature review and Table 1, shows the predicted future damage potentials for tree species based on their growth and development characteristics.

### 2.4 Statistics

Microsoft Excel (www.microsoft.com) and IBM SPSS Statistics Version 20 (www.ibm.com) were used. All data collected have been quality-checked, and outliers have been identified and removed if there was a scientific reason to do so. Descriptive summary statistics, regression analysis and the non-parametric Mann-Whitney U-test have been performed for statistically valid data sets such as damage to structures, if data sets were sufficiently large. Significant (p < 0.05) findings have been highlighted, where appropriate. The ability to conduct further statistical analyses of the data set was limited due to the small sample size for most species causing damage and the dynamic nature of the urban environment.

## 3 Results and discussion

### 3.1 General overview

A total of 536 mature trees were assessed in detail. After applying the criteria given above, the tree species percentage occurrence reduced accordingly. Table 2 shows a summary of the most frequently occurring trees and their corresponding damage recordings to key urban structures. The range of tree size has been limited by including only trees that have DBH entries of at least 10 cm to avoid skewing the data set towards small trees that might not survive. Furthermore, small and young trees have not yet developed sufficient size and strength to cause damage to the surrounding infrastructure. The fact that the standard deviations of DBH are high reflects the point that even mature trees are highly variable in size, which is natural. The application of the proposed methodology has been demonstrated in Table 2, Table 3, Table 4, Table 5, Table 6 as discussed in Sections 3.2–3.4 below.

### 3.2 Structural damage

The proportions of structures that were linked to damage from trees can be found in Table 2. Of the total 231 damaged structures observed, the following proportions expressed in percentages can be calculated: impermeable pavements (44%), permeable pavements (22%), kerbs (19%), retaining walls (5%), footpaths (4%), roads (3%) and walls (3%). No damage to buildings (0%) has been recorded. The patterns associated with damage linked to impermeable pavements compared to permeable pavements are in line with the findings by Randrup et al. (2003). This suggests the need for more retrofitting of robust SuDS techniques (Scholz and Uzomah, 2013). However, it is expected that the severity of damage will advance further with time.

No damage to buildings was recorded. This is possibly due to the fact that the assessment was only based on an external visual observation. An internal structural assessment may reveal damage to buildings. Moreover, most buildings have formidable foundations and may not be easily damaged as compared to road structures and pathways.

By dividing the number of a particular species causing damage by the number of the corresponding species occurrence, the proportion of damage caused by this species can be calculated from Table 2. In contrast to the control group (48% of *A. platanoides* that did not cause any damage), about 52% of all *A. platanoides* caused various kinds of damage (as detailed in Table 2) to urban structures determined by expert judgement. The proportions of the other species that caused damage were as follows: *Aesculus hippocastanum*, 59%; *Tilia platyphyllos* Scop. (large-leaved lime), 53%; *F. excelsior*, 45%; *A. pseudoplatanus*, 42%; *Tilia cordata* Mill. (small-leaved lime), 36%; *Fagus sylvatica* L. (common beech), 33%; *Betula pendula* L (silver birch), 32%; *P. avium*, 15%; and *Crataegus monogyna* L. (common hawthorn), 11%. The severity of corresponding damage was in the following order: moderate (66%); light (21%); and severe (13%).

Findings indicate that there were no obvious patterns of damage to structures. This could be attributed to relatively small sample sizes and complex processes such as differences in soil moisture content, various levels of structural compactions, and average distance of trees from structures. For example, trees are normally planted closer to permeable pavements, impermeable pavements and kerbs compared to roads.

In order to achieve maximum ecosystem service benefits, the most suitable trees that could be combined with SuDS are the one that (a) are as close to structure as possible; (b) have a large diameter; (c) cause the least or no damage; and (d) are readily available and desirable by residents. The closer trees are to the structures or residents, the more the effects are felt; e.g., reducing localised extreme temperatures. The greater a tree diameter, the more mature the tree is likely to be and, therefore, the more noticeable will be the tree benefits (Leuzinger et al., 2010). Trees linked to a low damage potential are usually preferred both for new construction or retrofitting of SuDS sites. Vegetation that is desirable by residents is usually associated with high aesthetic values such as mature and beautiful trees with a perceived rich character.

### 3.3 Damage to structures linked to tree diameter and distance

#### 3.3.1 Overview

**Fig. 2:**
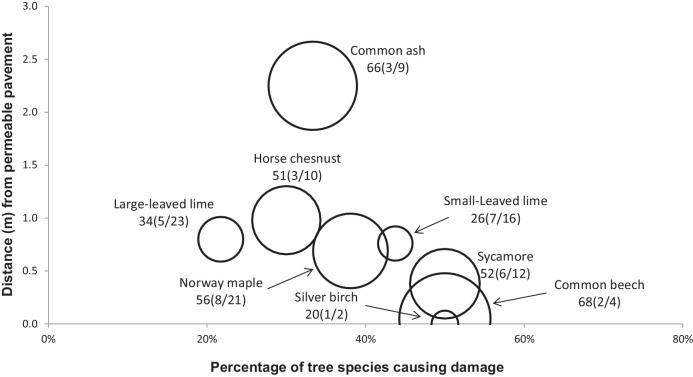
Relationships of tree diameters at breast height (DBH (cm); represented by circles), average distances of trees away from permeable pavements, and the proportion of trees within 10 m to these structures subjected to moderate to severe damage. Note: x(y/z), where x represents DBH and z indicates the number of the tree species out of which y trees caused damage.

**Fig. 3:**
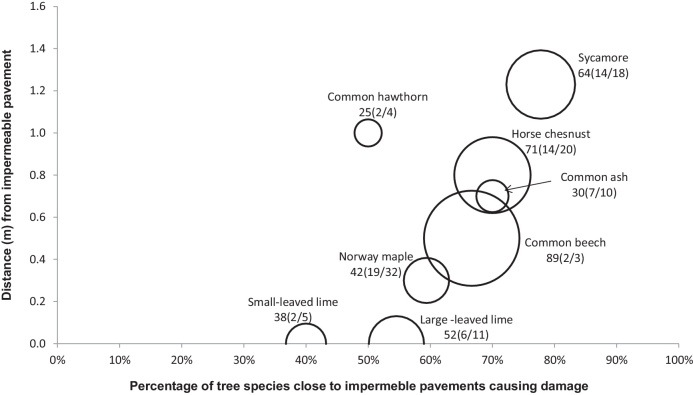
Relationships of tree diameters at breast height (DBH (cm); represented by circles), average distances of trees away from impermeable pavements, and the proportion of trees within 10 m to this structure subjected to moderate to severe damage. Note: x(y/z), where x represents DBH and z indicates the number of the tree species out of which y trees caused damage.

**Fig. 4:**
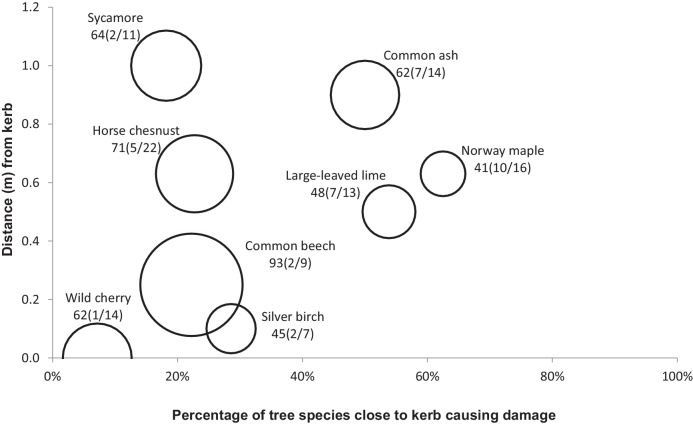
Relationships of tree diameters at breast height (DBH (cm); represented by circles), average distances of trees away from kerbs, and the proportion of trees within 10 m to these structures subjected to moderate to severe damage. Note: x(y/z), where x represents DBH and z indicates the number of the tree species out of which y trees caused damage.

**Fig. 5:**
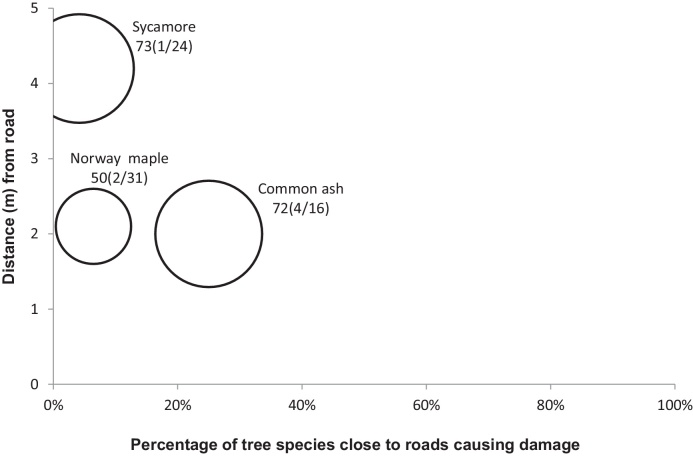
Relationships of tree diameters at breast height (DBH (cm); represented by circles), average distances of trees away from roads, and the proportion of trees within 10 m to these structures subjected to moderate to severe damage. Note: x(y/z), where x represents DBH and z indicates the number of the tree species out of which y trees caused damage.

**Fig. 6:**
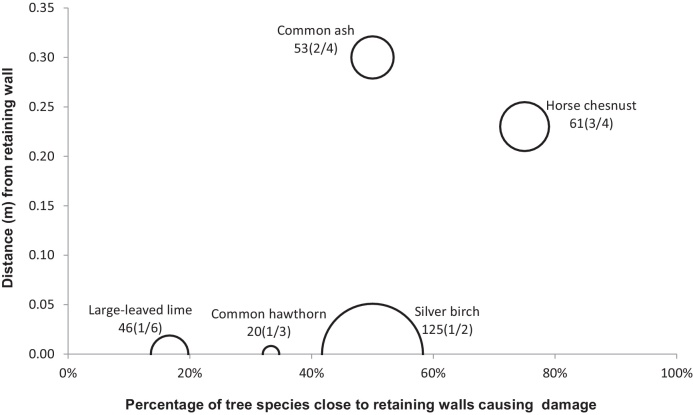
Relationships of tree diameters at breast height (DBH (cm); represented by circles), average distances of trees away from retaining walls, and the proportion of trees within 10 m to these structures subjected to moderate to severe damage. Note: x(y/z), where x represents DBH and z indicates the number of the tree species out of which y trees caused damage.

**Fig. 7:**
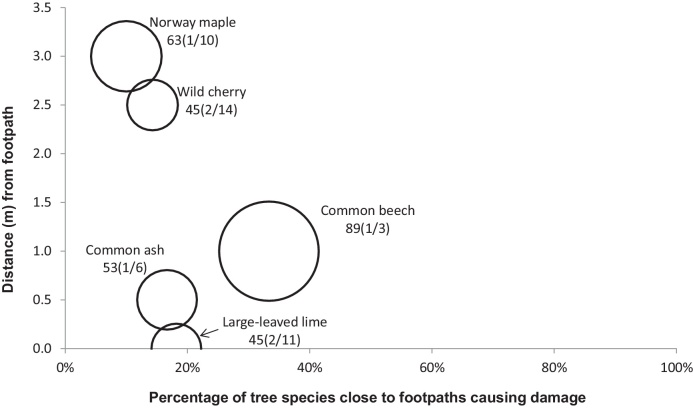
Relationships of tree diameters at breast height (DBH (cm); represented by circles), average distances of trees away from footpaths, and the proportion of trees within 10 m to these structures subjected to moderate to severe damage. Note: x(y/z), where x represents DBH and z indicates the number of the tree species out of which y trees caused damage.

**Fig. 8:**
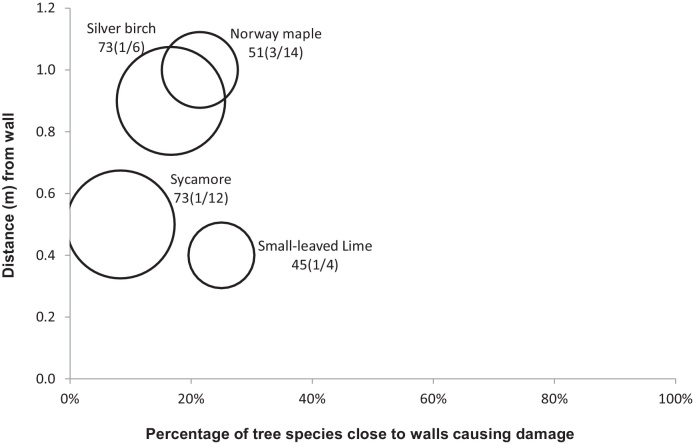
Relationships of tree diameters at breast height (DBH (cm); represented by circles), average distances of trees away from walls, and the proportion of trees within 10 m to these structures subjected to moderate to severe damage. Note: x(y/z), where x represents DBH and z indicates the number of the tree species out of which y trees caused damage.

Fig. 2, Fig. 3, Fig. 4, Fig. 5, Fig. 6, Fig. 7, Fig. 8 show the relationship of tree DBH, average distance of trees away from the structures, and the proportion of trees close to structures that caused moderate to severe damage. Note that only moderate and severe damage was considered, considering that the reason for light damage is often unclear. Apart from tree-related damage, other reasons for damage might be as important but only further destructive tests on site might reveal the main reason(s) for damage.

For x(y/z), where x represents the DBH (cm), which is also signified by the relative size (diameter) of the circle. The diameters expressed by circles give a visual indication of the maturity of the average tree species, which makes visual comparisons between trees easier. The entry z indicates the number of the tree species within 10 m of the structure, out of which *y* trees caused moderate to severe damage.

#### 3.3.2 Permeable pavement

For permeable pavements, most significant (p < 0.05) damage to permeable pavements was caused by trees located within 0–1.0 m away from a structure, except for those from *F. excelsior*. About 33% of *F. excelsior* located close to permeable pavements caused damage to these pavements if their average diameter was 66 cm and if their average distance was 2.3 m away from the permeable pavements (Fig. 2). The trees with the highest percentage of moderate and severe damage to permeable pavements (up to 50%) were *F. sylvatica*, *A. pseudoplatanus* and *B. pendula*. However, the corresponding sample sizes were rather small. The average distance of *F. sylvatica* and *B. pendula* to permeable pavements was 0 m, indicating that most of these two species were planted too close to the pavement. The average DBH of these trees was 68 cm and 20 cm, respectively (Fig. 3).

*Acer platanoides* caused the most overall damage (2 light, 4 moderate and 4 severe damage) to permeable pavements (Table 3), which was statistically significant (p < 0.05). The average diameter of the tree was 56 cm and the mean distance from the permeable pavements was 0.8 m. However, Fig. 3 shows a comparison for only moderate and severe damage. Seven out of sixteen *T. cordata* trees located close to permeable pavements caused major damage. The corresponding average tree diameter was 26 cm and the mean distance from the structures was 0.8 m. *Acer pseudoplatanus* caused six major damage incidents to permeable pavements. The average DBH of this tree was 52 cm and it was located about 0.4 m away from structures. *Tilia platyphyllos* caused five major damage occurrences to permeable pavements; its average diameter was 34 cm and the corresponding mean distance from structures was 0.8 m. For *F. excelsior*, although three major damage incidents to permeable pavements were recorded, the average distance from the structure was 2.3 m and the mean diameter was 66.1 cm, indicating that these were mature trees located further away from the structure, but still causing damage. Therefore, *F. excelsior* is not best suited close to permeable pavements.

The *F. sylvatica* assessed were mature trees with an average diameter of 68.1 cm and a mean distance of 0 m (i.e. touching the structures) from the building elements. On the other hand, using the metrics detailed in Table 1, *B. pendula* included in the analysis had not yet reached maturity. Their average DBH was 20.1 cm and they were planted too close to the structures. *Betula pendula* of this DBH was estimated to be about 20 years (see above).

#### 3.3.3 Impermeable pavement

Concerning impermeable pavements (Table 2), the majority of the damage occurred to these pavement structures (44%), which was statistically significant (p < 0.05). The reason for this is that impermeable pavements do not allow free circulation of moisture and air into and out of the pavement surface (Randrup et al., 2003; Day et al., 2010; Scholz, 2013). Because of this, pockets of moisture build-up below the surface of impermeable surfaces, causing the roots of trees below the impermeable surface to be attracted to these pockets of moisture, and thereby lifting-up of the corresponding pavement surface. This may have accounted for the relatively high number of damage to impermeable pavements.

For an impermeable pavement, the further away the tree (up to a distance of 1.4 m), the higher is the percentage of this tree causing damage irrespective of the tree DBH (Fig. 3). This is rather unexpected, considering that a large tree DBH is usually linked to large roots, which would cause damage at close range. However, the tree DBH variability is relatively small and the sample sizes are rather small as well. Regression analysis did not reveal any significant findings.

Wherever tree roots are deprived of air and moisture, they start to grow back towards the surface to obtain these resources. Morgenroth (2011) studied root distribution in relation to paved and normal surfaces in the top 30 cm of soil. He found that root abundance in the top 30 cm is greater in impermeable pavements than in normal soil.

This study revealed that the pavements of Greater Manchester roads consist of more impermeable pavements than permeable pavements. Considering the findings of Morgenroth (2011) and Viswanathan et al. (2011), the Greater Manchester case is more likely linked to the phenomenon of insufficient moisture in the compacted soil strata below the impermeable pavements, and the tendency of roots to remain close to the surface for oxygen and moisture availability. Hence, this is the reason for greater damage to impermeable pavements than permeable pavements. This phenomenon seems common where there are more impermeable pavements than porous surfaces. *Acer pseudoplatanus* caused the most damage to impermeable pavements (78%) from an average distance of 1.3 m and an average DBH of 64 cm.

#### 3.3.4 Kerb

Kerb damage comprised 19% of all recorded structural damage. *Acer platanoides* caused the most damage to kerbs (10 out of 16 trees were located close to kerbs) from an average distance of 0.6 m and with a mean DBH of 41 cm (Fig. 4). Similar to *A. platanoides* was the impact of *T. platyphyllos* (7 out of 13 nearby *T. platyphyllos*), *A. pseudoplatanus* (2 out of 11 surrounding trees) and *F. excelsior* (7 out of 14 surrounding trees) caused damage to kerbs from the furthest average distance of 1 m (Fig. 4).

Other trees that caused damage were less than 1 m from the kerb as shown in Fig. 4. *Prunus avium* was the best tree suitable for kerbs: only 1 in 14 trees caused moderate to severe damage (Fig. 4). However, most *P. avium* were very closely located (0 m) to kerbs, and their average DBH was 62 cm. This was closely followed by *F. sylvatica*. Although for *F. sylvatica* of an average DBH of 93 cm (indicating trees well-advanced in age) and an average distance of 0.23 m from kerbs, only 2 out of 9 trees caused moderate to severe damage to kerbs (Fig. 4). The worst tree to be located close to kerbs is *A. platanoides*. For trees of this species with an average DBH of 41 cm (indicating middle age) and located about 0.6 m from the kerbs, about 10 out of 16 *A. platanoides* caused moderate to severe damage to kerbs (Fig. 4).

#### 3.3.5 Other structures

The percentages of damage to roads and retaining walls were 3% each. Only three trees (*F. excelsior*, *A. platanoides* and *A. pseudoplatanus*) caused moderate to severe damage to roads (Fig. 5). Trees that caused damage to roads were located within an average distance of 2–5 m away from roads, indicating that the majority of them were planted close to the pavements.

Not many trees were found close to retaining walls. For *B. pendula* with a DBH of 125 cm and planted at an average distance to structures of close to 0 m, only 1 out of 2 trees caused moderate to severe damage (Fig. 6). *Aesculus hippocastanum* caused the most damage to retaining walls. Three out of four *A. hippocastanum* with an average DBH of 61 cm and located at a mean distance of 0.25 m away caused moderate to severe damage to retaining walls (Fig. 6).

### 3.4 Trees

*Acer platanoides* occurred the most frequently (17%) among other trees that were found in this survey (Fig. 1). Furthermore, *A. platanoides* caused the most severe damage to structures (Table 2 and Table 3). The damage done to structures by *A. platanoides* did not follow any particular pattern. In this survey, 38 out of 73 (52%) *A. platanoides* caused damage to various structures (Table 2). About 35% of all *A. platanoides* planted close to permeable pavements with an average DBH of 56 cm and an average distance of 0.75 m from the permeable pavements caused severe to moderate damage to the pavement structures. This average DBH represents maturing *A. platanoides*.

On average, *A. platanoides* caused more damage (42%) to impermeable pavements than to permeable pavements. These 42% of *A. platanoides* had an average DBH of 42 cm with an average distance of 0.3 m from impermeable pavements. This DBH represents *A. platanoides*, which are still in their relatively fast growth phase. This indicates that *A. platanoides* has a greater potential to cause more damage to impermeable pavements than to permeable pavements.

About 60% of *A. platanoides* with an average DBH of 41 cm caused severe to moderate damage to kerbs from an average distance of 0.6 m. *Acer platanoides* of this DBH are still in the growing stage, indicating a future potential to cause more damage to kerbs. It follows that *A. platanoides* should not be recommended for planting near kerbs, as it is ranked the least suitable tree for planting close to kerbs (Table 4).

Only 7% of *A. platanoides* with an average DBH of 50 cm caused severe and moderate damage to roads. Their average distance from roads was 2.0 m. Roads are normally well-compacted during construction to bear heavy traffic and haulage loads, and will therefore resist most damage from tree roots. Moreover, trees are normally at least 2.0 m located from roads, because of spaces for permeable or impermeable pavements and kerbs. Therefore, roads were linked to rather few damage incidents by tree roots.

There were no records of severe and moderate damage to retaining walls by *A. platanoides*. About 20% of *A. platanoides* planted close to walls of average DBH of 51 cm caused severe and moderate damage to these wall structures. Those that caused damage were placed at an average distance of 1.0 m from the walls.

About 10% of *A. platanoides* close to footpaths with an average DBH of 63 cm caused severe and moderate damage to footpaths. Those that caused damage were at an average distance of 3 m to the footpaths. Damage to footpaths by *A. platanoides* even at a distance of 3 m are possible, because the underlying soils at footpaths are not as compacted as those associated with other road structures. Despite that *A. platanoides* caused the most damage, and was also ranked the lowest in the potential for retrofitting (Table 5).

Concerning *T. platyphyllos*, most trees that caused damage to urban structures (for example, impermeable pavements, retaining walls and footpaths) were very closely located to these structures compared to other trees (Fig. 4, Fig. 6, Fig. 7). Based on the survey, there was no record of severe to moderate damage by *T. platyphyllos* to some structures such as roads and walls. About 20% of the *T. platyphyllos* planted close to permeable pavements caused severe and moderate damage to permeable pavements from an average distance of 0.7 m. The average DBH of *T. platyphyllos* that caused damage were 34 cm. *Tilia platyphyllos* of this diameter were considered as still being in their growing stage (Table 1). The older these trees become, the more severe the damage would be.

About 55% of the *T. platyphyllos* planted close to impermeable pavements caused severe to moderate damage to these structures. These trees were very close located to impermeable pavements as their average distance to the structures was 0 m at a mean DBH of 52 cm. About 25% of *T. platyphyllos* planted close to kerbs with an average DBH of 48 cm caused severe to moderate damage to kerb structures. Their average distance to the kerbs was 0.5 m. About 17% of *T. platyphyllos* planted close to retaining walls having an average DBH of 46 cm caused severe to moderate damage to these wall structures. Their average distance to the retaining walls was 0.0 m, indicating that they were very close (virtually touching) to these structures. Similarly, about 18% of *T. platyphyllos* planted close to footpaths having an average DBH of 46 cm caused severe to moderate damage to footpaths. Their average distance to footpaths was also 0.0 m.

When assessing the damage to structures caused by *T. platyphyllos* with the relative importance of these structures, *T. platyphyllos* came second in terms of choice (Table 5). Furthermore, *T. platyphyllos* did not rank high in terms of future potential for damage (Table 6).

*Fraxinus excelsior* caused severe to moderate damage to permeable pavements, impermeable pavements, kerbs, roads and retaining walls, but none to walls. Based on the results of this study, it can be inferred that the roots of *F. excelsior* can spread well beyond 2.0 m on the ground surface. About 35% of *F. excelsior* planted close to permeable pavements with an average DBH of 66 cm caused severe to moderate damage to permeable pavements from an average distance of 2.3 m. A *F. excelsior* tree of this DBH is considered to be fully grown (Dobrowolska et al., 2011). *Fraxinus excelsior* was the tree furthest away that caused damage to permeable pavements. This may be due to its great size.

About 70% of *F. excelsior* that were close to impermeable pavements caused severe to moderate damage to these pavement structures. The trees were of an average DBH of 30 cm and were located at a mean distance of 0.7 m from the impermeable pavements. *Fraxinus excelsior* trees of such DBH are considered to be young and developing, and are likely to cause more damage to any urban structures in the future.

About 50% of the *F. excelsior* trees that were located closely to kerbs (average distance of 0.9 m) caused severe to moderate damage. Their average DBH was 62 cm. Most of these trees could be considered as mature. About 25% of *F. excelsior* close to roads with an average DBH of 72 cm caused severe to moderate damage to these road structures. They were located at an average distance of 2.0 m to the roads. About 50% of the *F. excelsior* found close to retaining walls with an a mean DBH of 53 cm caused severe to moderate damage to the retaining walls. They were placed at an average distance of 0.3 m from the retaining walls. *Fraxinus excelsior* had the highest average distance from the retaining walls amongst other trees that caused damage to retaining walls. The percentage of *F. excelsior* that caused damage to footpaths was the least among damage to other structures. The percentage of the trees that caused damage to footpaths was about 18% with a mean DBH of 53 cm and located an average distance of 0.5 m from the footpaths.

*Fraxinus excelsior* ranked very high (8/10) in terms of potential for damage (Table 5), but ranked lower (5/10) in terms of potential for future damage. Most *F. excelsior* trees recorded in this survey were already mature, but reached less than half of their life span when compared with data shown in Table 1. Secondly, none of the *F. excelsior* trees were located very close to any structure. *Fraxinus excelsior* received average scores (51%) in terms of aesthetics in spring and summer, but very low scores (24%) for aesthetics in autumn.

*Acer pseudoplatanus* caused damage to structures, even if planted at distances that could be considered as far away from structures such as permeable pavements, impermeable pavements, kerbs and roads. However, there were no recorded damage by *A. pseudoplatanus* to footpaths and retaining walls. The average diameter of *A. pseudoplatanus* that caused damage to structures ranged from 52 cm for permeable pavements to 73 cm for both roads and walls. Findings indicated that 6 out of 12 *A. pseudoplatanus* (50%) with a mean DBH of 52 cm caused damage to permeable pavements at an average distance of 0.4 m (Fig. 2). *Acer pseudoplatanus* was the only tree that consistently caused damage from the furthest distance concerning kerbs, impermeable pavements and roads (Fig. 3, Fig. 4, Fig. 5). *Acer pseudoplatanus* was responsible for the most damage to impermeable pavements from the furthest average distance of 1.2 m with a mean DBH of 64 cm (Fig. 3).

Because of the potential to cause damage even from a relatively far distance, *A. pseudoplatanus* ranked very high (9/10) in the potential for damage (Table 5), and also ranked very high (10/10) in the potential for future damage (Table 6).

*Prunus avium* caused moderate to severe damage only to kerbs and footpaths. The corresponding damage to kerbs was the lowest (1/14 trees) among other trees. *Prunus avium* had an average DBH of 62 cm and were located very close (touching distance) to kerbs (Fig. 4). The number of *P. avium* that caused damage to footpaths was also very small (2/14). The DBH was 45 cm and the average distance from the footpaths was 2.5 m.

Concerning future damage, *P. avium* ranked third (Table 5), indicating that it is one of the preferred tree species when considering damage to structures. For predicted future damage potentials, it is ranking first (Table 6), highlighting that the damage from *P. avium* are unlikely to get worse compared to other trees. *Prunus avium* also scored very high (72%) concerning aesthetics in spring and summer, but low (36%) in autumn.

*Aesculus hippocastanum* caused moderate to severe damage to permeable pavements, impermeable pavements, kerbs and retaining walls, but none to roads, footpaths and walls. About 32% of *A. hippocastanum* were responsible for moderate to severe damage to these structures. Most *A. hippocastanum* that caused damage were mature in size with a mean DBH ranging from 51 to 71 cm (Fig. 2, Fig. 3, Fig. 4, Fig. 5, Fig. 6, Fig. 7, Fig. 8).

*Aesculus hippocastanum* was ranked as the second (2/10) best tree with regard to damage to structures, and ranked fourth best in the potential for future damage, because most of the assessed trees were already mature. However, *A. hippocastanum* leaves generally lead to considerable volumes of leaf litter on streets during autumn.

*Tilia cordata* caused moderate to severe damage to permeable pavements, impermeable pavements and walls, and no damage to kerbs, roads, retaining walls and footpaths (Fig. 2, Fig. 3, Fig. 4, Fig. 5, Fig. 6, Fig. 7, Fig. 8). Distances of *T. cordata* to structures were generally within a mean distance of 0 m (as for impermeable pavements) to 0.7 m (as for permeable pavements). Most *T. cordata* that caused damage could be classed as still being very young, since their average DBH were between 26 to 38 cm, compared with those of 146–200 cm for a mature *T. cordata* tree (Fig. 2, Fig. 3, Fig. 8 and Table 1).

*Tilia cordata* was responsible for damage already at young age (indicated by a small DBH). Therefore, this tree was considered to have a high potential to cause damage both in the present but particularly in the future.

*Betula pendula* caused moderate to severe damage to permeable pavements, kerbs, walls and retaining walls, but no harm to impermeable pavements, roads and footpaths. The DBH for *B. pendula* that caused damage varied widely: 20 cm for those trees near permeable pavements, 45 cm for those near kerbs, 73 cm for those near walls, and 125 cm for those near the retaining walls. Most *B. pendula* that caused harm were very close to the structures they damaged, except for those close to walls, which were located at an average of 0.9 m away from trees. Due to *B. pendula* being able to cause damage even at small DBH, it ranked very high in the potential for structural damage both at presence and in the future (Table 5 and Table 6).

*Crataegus monogyna* caused moderate to severe destruction to only impermeable pavements and retaining walls at an average DBH of 25 cm and 20 cm, respectively (Fig. 2, Fig. 3, Fig. 4, Fig. 5, Fig. 6, Fig. 7, Fig. 8), indicating that these were still relatively small trees. However, 2 out of 4 *C. monogyna* caused moderate to severe harm to impermeable pavements from an average distance of 1 m, while 1 out of 3 trees caused damage to retaining walls from a mean distance of 0 m (Fig. 3 and Fig. 6).

*Crataegus monogyna* ranked fifth in terms of potential for damage. This tree was located close to most structures, but damaged only two (Table 5). However, it ranked second in terms of potential for future damage (Table 6). The overall size of this tree may not increase significantly in the future due to its natural size, which is rather small compared to other trees such as *A. pseudoplatanus* (Table 1).

*Fagus sylvatica* was linked to moderate and severe destruction to permeable pavements, impermeable pavements, kerbs and footpaths, but no damage to roads, walls and retaining walls. The average DBH of most *F. sylvatica* trees causing damage was relatively large, ranging from 68 to 93 cm (Fig. 2, Fig. 3, Fig. 4 and Fig. 8), indicating that they are already large and mature (Table 1). In all cases of harm to structures, *F. sylvatica* trees appeared to be the largest trees in terms of DBH wherever they featured (Fig. 2, Fig. 3, Fig. 4 and Fig. 8).

### 3.5 Study limitations

The study has limitations due to the complex nature of the dynamic urban environment. In order to make sure that tree species, which have a good spread in Greater Manchester and that are typical for urban areas are well-reflected in this analysis, and also that recorded damage were actually caused by trees and not by other causes such as soil settlements due to unforeseen heavy traffic, the following criteria were applied:

(1) Tree species that had less than 10 occurrences in total were not included in the analysis;
(2) Tree species that occurred in less than five different sites were also discarded.
(3) All damage classed as ‘light’ was also not included in the detailed analysis to reduce the likelihood of making trees responsible for damage when in fact other causes of damage are potentially also likely. Alternative reasons for damage might be natural settling of structures, fatigue of old constructions and physical damage linked to road accidents.
(4) For the analysis of structural damage, only the structure types with at least ten damage reports linked to a specific tree species were considered to decrease the likelihood of high variability linked to small data sets to lead to spurious findings.

Furthermore, not all trees were located in areas where they had the opportunity to influence all categorised structures in the same manner; e.g., some of the trees were located, for example, in parks with no major structures (e.g., buildings and roads) around, and that may have reduced the proportion of trees that have caused damage to these types of structures. Therefore, it was important to base the study on a relatively large data set collected at random and to implement the above criteria limiting the risk of spurious findings.

## 4 Conclusions

Considering the damage to structures by trees, obvious patterns may not have been demonstrated due the small sample size for a given species combined with the variability of the growing conditions for each species and site. Nevertheless, based on the Greater Manchester case study, the ‘best trees’ (in terms of relatively low risk to infrastructure) to be recommended for temperate and oceanic climates are *T. platyphyllos*, *P. avium*, *C. monogyna*, *B. pendula*, *F. sylvatica*, *F. excelsior*, *A. pseudoplatanus* and *T. cordata*.

The project also concludes that impermeable pavements were subject to the highest number of damage from trees (44%), followed by permeable pavements and kerbs (22% and 19%, respectively). Trees planted close to impermeable pavements will cause more damage to the structure compared to those planted close to permeable pavements under the same conditions, which should be considered by town planners in the future.

Other structural damage to roads, retaining walls and houses ranged from 0 to 5%. These rather low figures can be explained by the high compaction of the underlying media during their construction. Planners should consider that the more compacted underlying materials are, the greater is the likelihood that tree roots will spread close to the surface, and thereby damaging roads and SuDS structures. Roots of trees planted in not compacted underlying soil media, for example, in parks, fields and footpaths, did not spread along the ground surface, but went deeper into the soil causing little or no damage to these structures. It follows that trees to be planted along streets in the future require more space and less compacted soil to reduce the risk of damage to nearby structures.

Considering that the proportion of trees causing damage to infrastructure is always relatively small, the corresponding sample size per species is also rather small. This makes a statistical analysis rather challenging. Therefore, the authors recommend to undertake further studies on a much larger scale, and to focus only on a specific group of trees. Such studies should also assess tree trunk and root flare developments to provide a better understanding of root growth and development, particularly under structures such as pavements as well as the interaction of roots and trunk flare with pavements.

## Declarations

### Author contribution statement

Miklas Scholz: Conceived and designed the experiments; Analyzed and interpreted the data; Wrote the paper.

Vincent Uzomah: Performed the experiments; Analyzed and interpreted the data; Wrote the paper.

Furat Al-Faraj: Contributed reagents, materials, analysis tools or data.

### Competing interest statement

The authors declare no conflict of interest.

### Funding statement

Vincent Uzomah was supported by The University of Salford.

### Additional information

No additional information is available for this paper.

## Figures and Tables

**Table 1:** Tree development characteristics (after Garden Centre (2015), Arbor Day Foundation (2015), British Hardwood Tree Nursery (2015), Pliûra and Heuertz (2003), and Defra (2007)).

Tree species	Maximum height (m)	Maximum diameter (cm)	Age to maturity (years)	Maximum age (years)	Early growth pattern				Growth rate	Root pattern	Best soil condition	Comments
					After 10 years of age		After 20 years of age					
					Height (m)	Crown (m)	Height (m)	Crown (m)				
*Acer platanoids* L.	15–30	150	40–50	250	8	4	13	7	Medium	–	Acidic, alkaline, loamy, moist, sandy, well-drained, wet and clay soils; some drought tolerance.	Rapid growth rate till maturity; tolerates pollution and other urban conditions well.
									35–60 cm/yr			
*Acer pseudoplatanus* L.	20–35	150	50–60	150–250	10	5	15	8	Fast	–	All soils; tolerates salt-laden soils.	Rapid growth rate till maturity.
									35–70 cm/yr			
									(Av.=50)			
*Fraxinus excelsior* L.	24–35	160	15–20	≤400	8	5	11	8	Medium	–	Prefers deep, moist and cool soil; tolerates pollution and exposed sites.	–
									35–60 cm/yr			
Prunus avium L.	5–20	120	3–7	20–90	8	5	14	7	Medium to fast	Requires deep soil	Prefers light and sandy soil, but grows also in moist and well-drained soil; Not drought-tolerant.	–
									35–60 cm/yr			
*Tilia platyphyllos* L.	24–28	146–200	35	500	8	3	12	8	Medium to fast	Deep roots	Any well-drained fertile soil; able to withstand shade and pollution.	–
									35–60 cm/yr			
*Aesculus hippocastanum* L.	28–35	150	20	300	8	4	11	8	Medium	–	Acidic, loamy, moist, rich, sandy, silty loam, well-drained and clay soils.	Rapid growth rate in the first 10 years
									35–60 cm/yr			
*Tilia cordata* L.	24–28	146–200	35	500	6	4	12	6	–	Deep roots	Good light loam	–
*Betula pendula* L.	15–25	30–150	50	50–100	8	3	18	4	Fast	–	Rich humus and raw soil of mountainside.	Rapid growth (50–60 cm/yr) in first 20 years.
									35–70 cm/yr			
*Crataegus monogyna* L.	≤12	30–100	–	100–150	4	3	8	5	Slow to medium	–	–	–
									30–60 cm/yr			
									(av.=40)			
*Fagus sylvatica* L.	15–18	190	18	150–200	4	4	14	7	Slow to medium	Does not need deep soil	Acidic, loamy, moist, sandy, well-drained and clay soils; prefers moist and well-drained soil, but has some drought tolerance.	Branches close to the ground
									30–60 cm/yr			

**Table 2:** Summary of the most frequently occurring trees and their corresponding damage recordings to key urban structures located in the studied square sites of 100 m × 100 m.

Species	Number of occurrence	Percentage occurrence (%)	Number of sites where each species is present	Number of trees that caused damage	Number of damage on permeable pavement	Number of damage on impermeable pavement	Number of damage on kerb	Number of damage on road	Number of damage on retaining wall	Number of damage on footpath	Number of damage on wall
*Acer platanoide* L.	73	20	24	38	10	29	12	2	0	3	3
*Acer pseudoplatanus* L.	50	13.7	22	21	9	19	3	1	0	0	1
*Fraxinus excelsior* L.	44	12	22	20	4	11	8	4	4	1	0
*Prunus avium* L.	40	10.9	19	6	4	0	1	0	0	2	0
*Tilia platyphyllos* L.	38	10.4	14	20	7	13	9	0	1	2	0
*Aesculus hippocastanum* L.	37	10.1	11	22	6	21	6	0	3	0	0
*Tilia Cordata* L.	28	7.7	14	10	8	2	2	0	0	0	1
*Betula pendula* L.	25	6.8	13	8	1	2	2	0	2	1	1
*Crataegus monogyna* L.	19	5.2	10	2	0	2	0	0	1	0	0
*Fagus sylvatica* L.	12	3.3	7	4	2	2	2	0	0	1	0
TOTAL	366	100%	156	151	51	101	45	7	11	10	6

*Note: Please note that some trees caused damages to more than one structure, and as such the addition of the number of damage to all the structures may exceed the number of species that cause damage.

**Table 3:** Tree damage to structures for trees that occurred ≥10 times and which were found in ≥5 different sites out of the 100 randomly selected sites in Greater Manchester.

Severity of damage	Number, distance and/or diameter	Statistic	*Acer platanoide* L.	*Acer pseudoplataneus* L.	*Fraxinus excelsior* L.	*Prunus avium* L.	*Tilia platyphyllos* L.	*Aesculus hippocastanum* L.	*Tilia cordata* L.	*Betula pendula* L.	*Crataegus monogyna* L.	*Fagus sylvatica* L.
Damage to permeable pavements												
Light	Number		2	3	1	3	2	1	1	0	0	0
Moderate	Number		4	6	3	1	5	5	7	0	0	2
	Distance (m) from structure	Mean	1	0	2	2	1	1	1	–	–	0
		Standard deviation	1.2	0.5	1.8	0	0.2	0.8	0.8	–	–	0.1
	Diameter (cm) at breast height	Mean	54	52	66	–	34	51	26	–	–	68
		Standard deviation	3.8	20.5	10.7	–	14.2	18.7	8.6	–	–	28.3
Severe	Number		4	0	0	0	0	0	0	1	0	0
	Distance (m) from structure	Mean	0	–	–	–	–	–	–	0	–	–
		Standard deviation	0	–	–	–	–	–	–	0	–	–
	Diameter (cm) at breast height	Mean	58	–	–	–	–	–	–	20	–	–
		Standard deviation	0.5	–	–	–	–	–	–	0	–	–
–												
Damage to impermeable pavements												
Light	Number		6	3	4	0	3	3	0	2	0	0
Moderate	Number		18	13	7	0	10	15	0	0	2	1
	Distance (m) from structure	Mean	0	1	1	–	0	1	–	–	1	1
		Standard deviation	1	1.4	0.2	–	0	0.2	–	–	0	0
	Diameter (cm) at breast height	Mean	41	57	25	–	52	70	–	–	25	89
		Standard deviation	21.5	22.7	8.5	–	8.1	20.5	–	–	0	0
Severe	Number		5	3	0	0	0	3	2	0	0	1
	Distance (m) from structure	Mean	0	1	–	–	–	1	0	–	–	1
		Standard deviation	0.1	1.4	–	–	–	0.3	0	–	–	0
	Diameter (cm) at breast height	Mean	48	73	–	–	–	77	38	–	–	89
		Standard deviation	6.8	24	–	–	–	12.1	0	–	–	0

Damage to kerbs												
Light	Number		3	1	1	0	2	2	2	0	0	0
Moderate	Number		6	2	5	1	6	4	0	2	0	0
	Distance (m) from structure	Mean	1	1	1	0	1	1	–	0	–	–
		Standard deviation	0.9	0	0.8	0	1	0.2	–	0	–	–
	Diameter (cm) at breast height	Mean	42	64	66	62	48	70	–	45	–	–
		Standard deviation	7.6	0	20.1	0	6.1	26.9	–	27.9	–	–
Severe	Number		3	0	2	0	1	0	0	0	0	2
	Distance (m) from structure	Mean	0	–	1	–	0	–	–	–	–	0
		Standard deviation	0.1	–	0.9	–	0	–	–	–	–	0.3
	Diameter (cm) at breast height	Mean	38	–	36	–	43	–	–	–	–	93
		Standard deviation	8.1	–	3.7	–	0	–	–	–	–	3.7

Damage to retaining walls												
Light	Number		0	0	0	0	0	0	0	1	0	0
Moderate	Number		0	0	3	0	1	3	0	1	1	0
	Distance (m) from structure	Mean	–	–	0	–	0	0	–	0	0	–
		Standard deviation	–	–	0.2	–	0	0.2	–	0	0	–
	Diameter (cm) at breast height	Mean	–	–	58	–	46	61	–	125	20	–
		Standard deviation	–	–	14.1	–	0	8.1	–	0	0	–
Severe	Number		0	0	1	0	0	0	0	0	0	0
	Distance (m) from structure	Mean	–	–	0	–	–	–	–	–	–	–
		Standard deviation	–	–	0	–	–	–	–	–	–	–
	Diameter (cm) at breast height	Mean	–	–	38	–	–	–	–	–	–	–
		Standard deviation	–	–	0	–	–	–	–	–	–	–

Damage to footpaths												
Light	Number		2	0	0	0	0	0	0	1	0	0
Moderate	Number		1	0	1	2	2	0	0	0	0	1
	Distance (m) from structure	Mean	3	–	1	3	0	–	–	–	–	1
		Standard deviation	0	–	0	0	0	–	–	–	–	0
	Diameter (cm) at breast height	Mean	63	–	53	49	45	–	–	–	–	89
		Standard deviation	0	–	0	20.8	0	–	–	–	–	0

Note: Trees that caused ≤10 structural damage in total and light damage entries were excluded from further analysis.

**Table 4:** Proportion of tree species that caused structural damage (trees causing damage/sum of trees causing and not causing damage).

Tree species	Impermeable pavements	Permeable pavements	Kerbs	Roads	Retaining walls	Footpaths	Buildings
*Acer platanoide* L.	19/32	8/21	10/16	2/31	0/0	1/10	0/8
*Acer pseudoplatanus* L.	14/18	6/12	2/11	1/24	0/4	0/13	0/2
*Fraxinus excelsior* L.	7/10	3/9	7/14	4/16	2/4	1/6	0/3
*Prunus avium* L.	0/6	1/15	1/14	0/13	0/2	2/14	0/2
*Tilia platyphyllos* L.	6/11	5/23	7/13	0/22	1/6	2/11	0/8
*Aesculus hippocastanum* L.	14/20	3/10	5/22	0/21	3/4	0/2	0/0
*Tilia cordata* L.	2/5	7/16	0/10	0/8	0/5	0/1	0/1
*Betula pendula* L.	1/14	1/2	2/7	0/12	1/2	0/5	0/3
*Crataegus monogyna* L.	2/4	0/0	0/4	0/4	1/3	0/3	0/3
*Fagus sylvatica* L.	2/3	2/4	2/9	0/3	0/1	1/3	0/3

**Table 5:** Presentation of relative tree rankings concerning the structural damage. The rankings indicate increases of ‘potential for damage’, where 1 represents the least potential for damage and where 10 represents the highest potential for damage. Weighting factors reflect the relative importance of structures based on civil engineering expert judgement by the authors.

Tree species	Impermeable pavements		Permeable pavements		Kerbs		Roads		Retaining walls		Footpaths		Buildings		Overall relative tree ranking	Best tree ranking
	(weight = 5)		(weight = 7)		(weight = 4)		(weight = 8)		(weight = 4)		(weight = 4)		(weight = 10)		(total WR)/total RR)	
	RR	WR	RR	WR	RR	WR	RR	WR	RR	WR	RR	WR	RR	WR		
*Acer platanoids* L.	6	30	5	35	10	40	3	24	NA	NA	10	40	1	10	5.11	4th
*Acer pseudoplatanus* L.	10	50	6	42	7	28	4	32	2	8	1	4	1	10	5.61	9th
*Fraxinus excelsior* L.	8	40	9	63	9	36	7	56	8	32	4	16	1	10	5.5	8th
*Prunus avium* L.	1	5	1	7	3	12	2	16	3	12	7	28	1	10	5	3rd
*Tilia platyphyllos* L.	4	20	2	14	8	32	1	8	4	16	2	8	1	10	4.91	2nd
*Aesculus hippocastanum* L.	7	35	4	28	5	20	1	8	9	36	8	32	NA	NA	4.68	1st
*Tilia cordata* L.	3	15	7	49	1	4	5	40	1	4	9	36	1	10	5.85	10th
*Betula pendula* L.	2	10	8	56	6	24	2	16	7	28	3	12	1	10	5.38	6th
*Crataegus monogyna* L.	9	45	NA	NA	2	8	6	48	5	20	6	24	1	10	5.34	5th
*Fagus sylvatica* L.	5	25	3	21	4	16	6	48	6	24	5	20	1	10	5.47	7th

RR, relative ranking; WR, weighted ranking; NA, not applicable.

**Table 6:** Predicted future damage potentials for tree species based on their growth and development characteristics. Relative rankings (RR) of the ‘potential for future damage’, where 1 represents least potential for damage, and 10 represents highest potential for damage. Weighting factors reflect the relative importance of structures based on civil engineering expert judgement by the authors.

Tree species	Impermeable pavements (weight = **5**)		Permeable pavements (weight = **7**)		Kerbs (weight = **4**)		Roads (weight = **8**)		Retaining walls (weight = **4**)		Footpaths (weight = **4**)		Buildings (weight = **10**)		Overall relative ranking (total WR/total RR)	Best tree ranking
	RR	WR	RR	WR	RR	WR	RR	WR	RR	WR	RR	WR	RR	WR		
*Acer platanoids* L.	8	40	3	21	5	20	3	24	–	–	2	8	–	–	5.38	7th
*Acer pseudoplatanus* L.	5	25	5	35	1	4	1	8	–	–	–	–	–	–	6	10th
*Fraxinus excelsior* L.	7	35	1	7	2	8	2	16	3	12	4	16	–	–	4.95	5th
*Prunus avium* L.	1	5	–	–	7	28	–	–	–	–	1	4	–	–	4.11	1st
*Tilia platyphyllos*	10	50	4	28	6	24	–	–	4	16	5	20	–	–	4.76	3rd
*Aesculus hippocastanum* L.	4	20	2	14	3	12	–	–	2	8	–	–	–	–	4.91	4th
*Tilia Cordata* L.	9	45	6	42	–	–	–	–	–		–	–	–	–	5.8	9th
*Betula pendula* L.	3	15	8	56	8	32	–	–	1	4	–	–	–	–	5.35	6th
*Crataegus monogyna* L.	6	30	NA	–	–	–	–	–	5	20	–	–	–	–	4.55	2nd
*Fagus sylvatica* L.	2	10	7	49	4	16	–	–	–	–	3	12	–	–	5.44	8th

WR, weighted ranking; NA, not applicable.
