# Erratum to “Potential tree species for use in urban areas in temperate and oceanic climates”

**DOI:** 10.1016/j.heliyon.2016.e00185

**Published:** 2016-11-29

**Authors:** Miklas Scholz, Vincent C. Uzomah, Furat A.M. Al-Faraj

**Affiliations:** a Division of Water Resources Engineering, Faculty of Engineering, Lund University, P.O. Box 118, 22100 Lund, Sweden; b Civil Engineering Research Group, School of Computing, Science and Engineering, The University of Salford, Newton Building, Salford M5 4WT, England, United Kingdom

## Erratum

In the original published version of this article, the numerical values in Table 4 were inadvertently converted into dates. The publisher apologizes for the error. Both the HTML and PDF versions of the article have been updated to correct the error.

